# Exploring the Influence of Career Decision Self-Efficacy on Adjustment Challenges in Psychiatric Nursing Students: A Cross-Sectional Insight

**DOI:** 10.62641/aep.v53i1.1910

**Published:** 2025-01-05

**Authors:** Hui Li, Yanqing Wang, Rui Zhang, Cuicui Sun

**Affiliations:** ^1^Department of Basic Medicine, Medical School of Zhengzhou SIAS University, 451100 Zhengzhou, Henan, China; ^2^Operating Room, Shanxi Jincheng People's Hospital, 048000 Jincheng, Shanxi, China; ^3^Nursing School, Shijiazhuang Medical College, 050000 Shijiazhuang, Hebei, China

**Keywords:** psychiatry, nursing student, poor adaptation, career decision-making, sense of self-efficacy

## Abstract

**Objective:**

To explore the relationship between Career Decision-Making Self-Efficacy and maladjustment among psychiatric nursing students.

**Method:**

The results of baseline data, Mental Health Knowledge Questionnaire, Career Decision-Making Self-Efficacy scale and Clinical Practice Maladjustment Questionnaire of psychiatric nursing students from January 2022 to August 2023 were obtained from our hospital, and the correlation of scores was examined through Pearson correlation analysis. The factors affecting psychiatric nursing students' maladjustment were analyzed through logistic regression analysis.

**Results:**

A total of 286 psychiatric nursing students were included in this study. The total score of all students of Mental Health Law Knowledge Questionnaire score was 21.30 ± 5.28, the total score of Career Decision-Making Self-Efficacy scale was 132.90 ± 13.36 and the total score of Clinical Practice Maladjustment Questionnaire was 102.85 ± 9.81. Positive correlations were found among the Mental Health Knowledge Questionnaire score and the Career Decision-Making Self-Efficacy scale and Clinical Practice Maladjustment Questionnaire (r = 0.550, 0.602, *p* < 0.05). Similarly, a positive correlation was found between the Career Decision-Making Self-Efficacy scale and Clinical Practice Maladjustment Questionnaire (r = 0.639, *p* < 0.05). Personality, school performance, Mental Health Knowledge Questionnaire score and Career Decision-Making Self-Efficacy Scale score were the main factors affecting clinical practice inadaptability of psychiatric nursing students, and the odds ratio (OR) values were higher than 1.

**Conclusion:**

Psychiatric nursing students experienced maladjustment during clinical practice, and Career Decision-Making Self-Efficacy was the main influencing factor.

## Introduction

Psychiatric departments are one of the key sections in hospitals, and most patients admitted to these departments are psychiatric patients with special conditions, who need a high level of nursing care. Nursing students can acquire knowledge and skills and develop work ethics through clinical practice [1]. A survey [2] has shown that some nursing students experience different degrees of adjustment disorder, also known as practice maladjustment, during clinical practice. Notably, according to the relevant study [3], approximately 48.9% of nursing students experience maladjustment. At the same time, adaptation problems can occur in the early, middle and late periods of internship, which will cause nursing students to lose motivation to work, cause physical and mental health problems, cause tension and alienation in the relationship between nursing students and colleagues, patients and family members, affect work atmosphere and teamwork [4, 5]. Because psychiatric nursing students have to deal with the special group of mentally ill patients and work in a state of high tension and concentration every day [6, 7], they are more likely to suffer from maladaptive phenomena. Maladjustment is an important issue for psychiatric nursing students and is directly related to their own health and career development.

Self-efficacy of career decision-making refers to the assessment and belief that one must have the ability to complete tasks during career decision-making [8].

The occurrence of maladaptation is closely related to the psychological qualities and career decisions of nursing students themselves. Career decision self-efficacy refers to an individual’s confidence and belief in their ability to successfully achieve goals in the career decision-making process. High self-efficacy can help nursing students better cope with challenges and pressures in their career development, thereby reducing the risk of maladaptation. A study has shown that career decision self-efficacy is closely related to individuals’ coping abilities when facing career challenges. For example, Liu *et al*. [9] found that career decision self-efficacy can predict individuals’ performance and outcomes in the career decision-making process. Research has shown that vocational education skills significantly contribute to improving vocational adaptability, with college students’ career decision self-efficacy playing a mediating role. A study involving 205 undergraduate students indicated that self-efficacy in career decision-making plays a mediating role in vocational adaptability, demonstrating a strong linear correlation between self-efficacy in career decision-making and vocational adaptability [10]. Most studies have shown that in different professions, there is a good correlation between self-efficacy in career decision-making and the ability to adapt to different occupational situations [11, 12]. Therefore, based on existing literature, we hypothesize that there is a significant negative correlation between career decision self-efficacy and maladaptation among psychiatric nursing students. Psychiatric nursing students with higher career decision self-efficacy are more likely to cope with various challenges in clinical practice, thereby reducing the occurrence of maladaptation.

Although some studies have explored the relationship between career decision self-efficacy and maladaptation, research specifically targeting psychiatric nursing students remains limited, especially in China. Therefore, this study aims to further investigate the relationship between career decision self-efficacy and maladaptation among psychiatric nursing students through a cross-sectional study, with the hope of providing theoretical guidance and practical reference for improving the vocational adaptability of psychiatric nursing students and promoting their career development.

## Materials and Methods

### Research Object

This study employed a cross-sectional research design. Continuous fixed-point sampling was utilized to collect data from psychiatric nursing students in our institution between January 2022 and August 2023. Inclusion criteria: (1) all nursing students in the department of psychiatry; (2) females; (3) nurses who successfully completed their studies in the school; (4) full-time junior college students; (5) nurses at least 18 years old. Exclusion criteria: (1) Males; (2) students were unable to abide by rules and regulations during the internship; (3) students who requested time off during internship; (4) students who were unable to complete the survey because of mental illness. This study was conducted in accordance with the Declaration of Helsinki, the protocol was approved by the Ethics Committee of Nursing School, Shijiazhuang Medical College (approval number: NO. 20211209), and informed consent was obtained from the participants.

### Method

We obtained basic information and questionnaire survey results of psychiatric nursing students from the hospital electronic information system. Referring to previous literature [13, 14], we selected several basic information: gender, age, education level, household registration, personality characteristics and school performance. Personality characteristics were assessed using the E scale in the Eysenck Personality Questionnaire. It had 21 items and mainly measures extroversion or introversion. >15 points were extroverts, <8 points were introversions, and 8–15 points were ambiverts. The Cronbach’s α coefficient of scale was 0.894 [15]. Performance in school was determined based on the final exam scores, average score of no less than 85 in each subject was considered good, 75–84 was considered good, and <75 was considered average or below. The destination after graduation was collected through post-graduation destination questionnaire online. It mainly included engaging in nursing work, taking the postgraduate examination, being unemployed or changing careers, etc. Nursing students chose based on their actual situation. Taking postgraduate examination, being unemployed or changing careers were classified into other categories.

The 31-item Mental Health Knowledge Questionnaire of this research used the Mental Health Law Knowledge Questionnaire compiled by Lan [16] according to the Mental Health Law of the People’s Republic of China. The scoring system assigned a value of 0–1 to each topic, and the total score ranged from 0 to 38. High scores reflected high level of understanding regarding mental health laws. If more than 23 items were answered correctly, the awareness score was deemed to be a passing grade, that is, the awareness is good; otherwise, the awareness score represented a failing grade [16].

The Career Decision-Making Self-Efficacy scale of this research was compiled by Peng and Long [17] on the basis of the Career Decision-Making Self-Efficacy scale. This scale involved 5 items including self-evaluation, information collection, goal selection, planning, and problem solving, totaling 39 items. The scale used a five-point Likert scale ranging from not at all confident to completely confident on a scale of 1–5. Total scores ranged from 39 to 195, and a total score of ≥108 points indicated that nursing students have strong confidence in career decisions. The Cronbach’s α coefficient of the scale is 0.937.

The maladaptive clinical practice questionnaire was compiled by O’Lynn [18]. The questionnaire consisted of 28 items that covered four aspects: interpersonal relationship, cognitive aspect, behavioural performance and emotional aspects. The five-point Likert grading method was employed, ranging from complete agreement to complete disagreement, with a total score of 1–5, and a total score of less than 95 indicates maladaptation.

### Statistical Methods

SPSS25.0 statistical software (IBM Corp., Armonk, NY, USA) was used in the analysis of relevant data, and the count data were expressed as [n (%)]. χ^2^ test was used. Measurement data were expressed as ($\bar{\chi }$ ± s), and *t*-test was used. Pearson correlation analysis was used in the analysis of the correlation among the Mental Health Knowledge Questionnaire, Career Decision-Making Self-Efficacy Scale and Clinical Practice Maladjustment Questionnaire scores. The nursing students were categorized into well-adjusted and maladjusted groups. The main factors affecting psychiatric nursing students’ maladjustment were explored through binary logistic regression analysis. The receiver operating characteristic curve (ROC) was drawn, and the predictive sensitivity, specificity, area under the curve (AUC) and Youden index of each factor were analysed for the identification of the optimal predictive cutoff value. The predictive value of each factor for nursing students’ maladjustment was observed. *p* < 0.05 was considered statistically significant.

## Results

### Flowchart of the Study Population

**Fig. 1.**
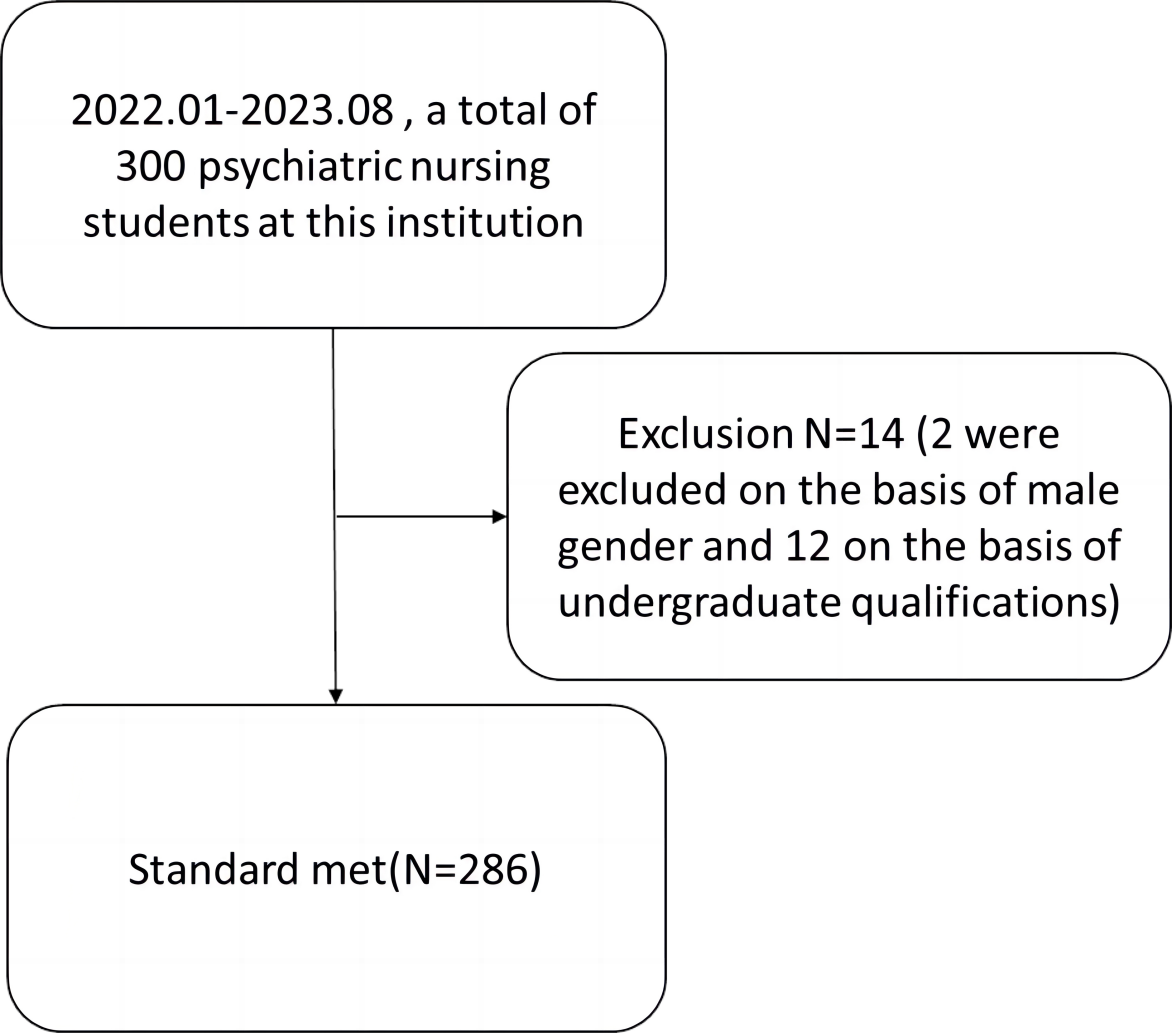
**Flowchart of the study population**.

From January 2022 to August 2023, there were a total of 300 psychiatric internship nursing students in our hospital. After excluding 14 students who did not meet the criteria, a total of 286 students were finally included, see Fig. 1.

### Survey Results of 286 Psychiatric Nursing Students 

The total scores of Mental Health Knowledge Questionnaire, Career Decision-Making Self-Efficacy scale and Clinical Practice Maladjustment Questionnaire were 21.30 ± 5.28, 132.90 ± 13.36 and 102.85 ± 9.81, respectively of the 286 psychiatric nursing students. The scores of each dimension are shown in Tables 1,2,3.

**Table 1.** **Correct response rate of Mental Health Knowledge Questionnaire among 286 psychiatric nursing students**.

Project		Correct response rate (%)	Project	Correct response rate (%)
(1) Implementation time		52.44	(11) Treatment of patients with mental disorders by medical personnel	93.71
(2) Guardian settings		89.86	(12) Scope of practice in psychological counselling	58.04
(3) Prevention obligations of the employer			(13) Confidentiality of psychological counselling	90.91
	(a) Create a work environment	91.61	(14) Diagnosis of maladjustment	60.49
	(b) Focus on mental health	90.91	(15) Medical evaluation for mental disorders	71.33
	(c) Health education	91.61	(16) Principles of diagnosis of suspected patients	76.92
(4) School prevention duty			(17) Diagnostic principles for admitting suspected patients	73.78
	(a) Carry out knowledge education	97.91	(18) Principles for the diagnosis of mental disorders	79.72
	(b) Pay attention to teachers’ mental health	91.61	(19) Principle of involuntary hospitalisation	71.33
	(c) Provide psychological assistance	90.56	(20) Involuntary admission without the consent of a guardian	37.06
	(d) Communicate with parents about psychological status	94.41	(21) Diagnosis and identification in emergency situations	82.17
(5) The counselor handled the patient		40.21	(22) Restraint and isolation measures	83.22
(6) Usual patients		63.64	(23) Provisions for psychosurgery	52.10
(7) Emergency patients			(24) Provision of patient communication and access rights	70.63
	(a) Close relative	69.93	(25) Provisions for discharge of voluntarily hospitalised patients	48.25
	(b) Work unit	63.64	(26) Provisions for discharge of involuntarily hospitalised patients	68.18
	(c) Local public security organs	90.21	(27) Regulations on medical record access and reproduction	58.04
(8) Duration of record keeping for mental disorders		39.16	(28) The patient must not be denied treatment for other diseases	63.60
(9) Psychological assistance in emergency plans		94.41	(29) Treatment location for psychotherapy	27.27
(10) Mental health		76.92	(30) Scope of practice in psychotherapy	49.30
			(31) Applicable provisions of law for violation by patients	61.19
Total score		21.30 ± 5.28		

**Table 2.** **Dimension scores and total scores of Career Decision-Making Self-Efficacy scale of 286 psychiatric nursing students (n = 286)**.

Career Decision-Making Self-Efficacy scale	Number of items (pieces)	Score ($\bar{\chi }$ ± s, score)
Total score	39	132.90 ± 13.36
Self-assessment	6	20.45 ± 4.80
Gather information	9	30.00 ± 6.28
Choose a goal	9	30.94 ± 6.45
Make a plan	8	27.46 ± 6.34
Solve a problem	7	24.05 ± 5.96

**Table 3.** **Dimension and total scores of Clinical Practice Maladjustment Questionnaire of 286 psychiatric nursing students (n = 286)**.

Clinical Practice Maladjustment Questionnaire	Number of items (pieces)	Score ($\bar{\chi }$ ± s, score)
Total score	28	102.85 ± 9.81
Interpersonal relationships	9	34.35 ± 5.92
Aspects of cognition	9	32.02 ± 6.38
Performance of behaviour	5	19.06 ± 3.77
The emotional aspect	5	17.42 ± 3.59

### Comparison of Clinical Practice Maladjustment Questionnaire Scores of Psychiatric Nursing Students with Different Characteristics in Psychiatric Department

No significant difference in Clinical Practice Maladjustment Questionnaire score was found among psychiatric nursing students in relation to age, household registration and post-graduation destinations (*p* > 0.05). There is significant difference in Clinical Practice Maladjustment Questionnaire score among psychiatric nursing students in terms of personality and school performance (*p* < 0.05), see Table 4.

**Table 4.** **Comparison of Clinical Practice Maladjustment Questionnaire scores of psychiatric nursing students with different characteristics ($\bar{\chi }$ ± s, score)**.

Project		Number of cases	Clinical Practice Maladjustment Questionnaire score	*t*/*F*	*p*
Age				0.630	0.529
	18–22 years old	204	102.62 ± 10.23		
	>22 years old	82	103.43 ± 8.72		
Household registration				0.632	0.528
	Rural areas	150	102.50 ± 10.11		
	Cities	136	103.24 ± 9.49		
Type of personality				11.425	<0.001
	Extroversion	108	105.27 ± 8.42		
	Ambiverts	80	104.05 ± 9.38		
	Introversion	98	99.20 ± 10.58		
School grades*				25.483	<0.001
	Good	50	108.14 ± 12.88		
	Better off	90	105.87 ± 7.34		
	Medium and below	146	99.18 ± 8.44		
Where to go after graduation				1.888	0.060
	Be a nurse	236	103.35 ± 9.31		
	Other^#^	50	100.48 ± 11.72		
Mental Health Knowledge Questionnaire score				2.269	0.024
	<20	79	100.73 ± 10.13		
	>20	207	103.66 ± 9.59		
Career Decision-Making Self-Efficacy scale scores				2.954	0.003
	<120	75	100.01 ± 9.10		
	>120	211	103.86 ± 9.87		

Note: ^*^ Average score at least 85 points is good, 75–84 points is better, <75 points is medium and below; # includes postgraduate entrance examination, unemployment or career change.

### Correlation Analysis of Mental Health Knowledge Questionnaire, Career Decision-Making Self-Efficacy Scale and Clinical Practice Maladjustment Questionnaire Scores among Psychiatric Nursing Students

The Mental Health Knowledge Questionnaire scores of psychiatric nursing students were significantly positively correlated with their Career Decision-Making Self-Efficacy scale and Clinical Practice Maladjustment Questionnaire scores (r = 0.550, 0.602; *p* < 0.05), and a positive correlation was found between the Career Decision-Making Self-Efficacy scale and Clinical Practice Maladjustment Questionnaire scores (r = 0.639; *p* < 0.05) (Table 5).

**Table 5.** **Correlation analysis of Mental Health Knowledge Questionnaire, Career Decision-Making Self-Efficacy Scale and Clinical Practice Maladjustment Questionnaire Scores among psychiatric nursing students**.

*r/p*	Mental Health Knowledge Questionnaire	Career Decision-Making Self-Efficacy scale	Clinical Practice Maladjustment Questionnaire scores
Mental Health Knowledge Questionnaire	-	r = 0.550	r = 0.602
		*p* = 0.018	*p* = 0.005
Career Decision-Making Self-Efficacy scale	-	-	r = 0.639
			*p* = 0.023

### Multivariate Analysis of Influencing Factors of Clinical Practice Maladjustment of Psychiatric Nursing Students

Logistic regression analysis was performed on the indicators that showed differences. Personality type, school performance, Mental Health Knowledge Questionnaire score and Career Decision-Making Self-Efficacy scale score were the main factors affecting psychiatric nursing students’ maladjustment during clinical practice, with odds ratio (OR) values greater than 1 (Table 6).

**Table 6.** **Multivariate analysis of influencing psychiatric nursing students’ maladjustment to clinical practice**.

Influencing factors	β value	SE	*p*	Wald value	Odds ratio (OR) value	95% confidence interval (CI)
Type of personality	0.496	0.162	0.002	9.374	1.642	1.195–2.256
School grades	0.556	0.135	0.001	16.962	1.744	1.339–2.272
Mental Health Knowledge Questionnaire score	0.533	0.180	0.003	8.768	1.704	1.197–2.425
Career Decision-Making Self-Efficacy scale scores	0.581	0.105	0.001	30.618	1.788	1.455–2.197

SE, standard error.

### Predictive Value of Each Factor for Clinical Maladjustment

The maladjustment in clinical practice was considered as the dependent variable, and the personality characteristics, school performance, score of mental health knowledge, and career decision-making self-efficacy were took as independent variables. The results of ROC curve analysis showed that the AUC of career decision-making self-efficacy was the largest (0.758), and the Youden index was 0.446, indicating higher diagnostic value, as shown in Table 7.

**Table 7.** **Analysis of the predictive value of each factor for clinical practice maladjustment**.

Characteristics	AUC value	Sensibility	Specificity	Yoden index	Optimal threshold value	95% CI	*p* value
Type of personality	0.515	0.508	0.862	0.370	135.00	0.456–0.574	<0.001
School grades	0.538	0.659	0.732	0.391	110.00	0.489–0.587	<0.001
Score of mental health knowledge	0.716	0.632	0.714	0.346	20.00	0.685–0.747	<0.001
Career Decision-Making Self-Efficacy scale score	0.758	0.664	0.782	0.446	107.00	0.715–0.801	<0.001

AUC, area under the curve.

## Discussion

### Career Decision of Psychiatric Nursing Students is Related to the Mastery of Mental Health Knowledge, Self-Efficacy and Adaptability in Clinical Practice

According to relevant research [19], the level of knowledge mastery of nursing students is directly correlated with their adaptability to the whole practice process. The total score of Mental Health Knowledge Questionnaire of 286 psychiatric nursing students was 21.30 ± 5.28, suggesting that the psychiatric nursing students had above-average mental health law knowledge. The reason may be the hospital’s diversified teaching methods which considered the needs of nursing students, which improved their mastery of theoretical knowledge. According to relevant research [20], many nursing students struggle to adapt to complex wording environments and exhibit varying levels of psychological, emotional and behavioural dysfunction. The total score of the Career Decision-Making Self-Efficacy scale of 286 psychiatric nursing students was 132.90 ± 13.36, which was slightly higher than the results of clinical reports [21]. This result indicated that the Career Decision-Making Self-Efficacy of nursing students was in the upper level, reflecting that nursing students had sufficient preparation and high self-confidence before practice. These characteristics may be related to the fact that our hospital’s teaching methods based on clinical needs are conducive to improving the self-efficacy of nursing students [22]. Nursing students demonstrated high proficiency in working goal selection and information collection, as indicated by their high scores for these two aspects. The reason may be the rapid development of network technology and improvements in nursing students’ ability to share professional information resources [23]. The low self-evaluation score may be attributed to the extremely high goals of nursing students at the beginning of clinical practice and lack of opportunity to present themselves [24]. A relevant study [25] has shown that the first 3 months of internship is the best period for nursing students to develop their concept, professional quality and clinical practice behaviour, but they are prone to maladaptation. Among 286 nursing students, the total score of Clinical Practice Maladjustment Questionnaire was 102.85 ± 9.81, which was slightly higher than the clinical report results [26]. The reason may be related to the close combination of theory, operation and practice post through active mobilisation before practice and different forms of practice teaching in our hospital. The scores of interpersonal relationship and social interaction were high possibly because of the communication skill training in the process of treatment in our hospital, which can improve nursing students’ communication skills. However, the low score of emotion may be due to the inability of nursing students to adjust their own state in the early stage of internship and increased tendency to show negative emotions [27, 28, 29].

### Influencing Factors of Maladjustment of Nursing Students in Psychiatric Department during Clinical Practice

#### Personality Characteristics, School Performance, and Degree of Mastery of Mental Health Knowledge

Personality type, school performance, and mastery of mental health knowledge were the main factors affecting psychiatric nursing students’ clinical practice maladjustment, and the OR value was greater than 1. All factors had high predictive value for clinical practice maladjustment. A study [30] believed that nursing students who are extroverted and ideal in school grades, and have a high degree of knowledge mastery exhibit strong adaptability in clinical practice. The reason is that extroverted nursing students can actively communicate with others, adjust their own state and actively integrate into a new environment. Nursing students with good performance in school and high degree of knowledge mastery have a solid theoretical foundation for clinical practice, are likely competent and quickly adapt to new environments [31]. Hospital managers should effectively cultivate the mentality of nursing students and provide support to nursing students with introverted personalities, poor academic performance and low level of knowledge [32, 33, 34, 35].

#### Career Decision-Making Self-Efficacy

The novelty of this study is determining the ROC curve of the Career Decision-Making Self-Efficacy scale score as a predictor of clinical practice maladjustment. The AUC was the main indicator for evaluating a predictive value, which ranged from 0.5 and 1.0. When AUC >0.5, the diagnostic effect improved as the AUC approached 1. Youden’s coefficient, also known as the accuracy index, is a parameter for evaluating the validity of screening tests. It is the sum of sensitivity and specificity minus 1. Locating an optimal threshold value in an ROC curve result in the identification of an optimal classification effect and fulfilment of practical application requirements and facilitate the assessment of sensitivity and specificity. The predictive sensitivity and specificity of Career Decision-Making Self-Efficacy scale scores for clinical practice maladjustment were 0.664 and 0.782, respectively, the Youden index was 0.446, the optimal threshold was 107.00 and the AUC value was 0.758, which were higher than those in clinical reports [36]. Career Decision-Making Self-Efficacy exhibits a high predictive value for clinical practice maladjustment. Nursing students with a high level of Career Decision-Making Self-Efficacy had high self-assessment ratings and confidence in completing internship tasks and achieving goals. These attributes can have a positive effect on their career development. In addition, the nursing profession has a high employment rate, and thus nursing students can obtain social resources and information, achieve sufficient understanding of clinical work and thus effectively adapt to new working environments [37, 38]. Additionally, support and care from schools, hospitals and families enhance the Career Decision-Making Self-Efficacy of nursing students and ensure the stable and healthy development of their nursing careers [39, 40, 41].

Individuals with mental illnesses may exhibit varying levels psychological abnormalities. They may commit suicide, injure themselves, hurt people and destroy things. These actions not only endanger themselves and their families but also affect their social security. The fundamental principle of mental patient care is to respect a patient’s personality and rights, particularly by providing sympathy and care. This form of care involves comprehending patients’ psychological state and social environments and their illnesses for the alleviation of their physical and mental suffering. This principle is the basis for establishing good nurse–patient relationships and the key to achieving optimal nursing outcomes. The career decisions and self-efficacy of psychiatric nursing students may influence their experiences and satisfaction that they gain from communicating and interacting with patients. In addition, the career decision-making and self-efficacy of psychiatric nursing students may affect the daily management and treatment of psychiatric patients. Therefore, schools and hospitals should provide opportunities to psychiatric nursing students to exercise and fully develop their potential and should focus on cultivating career decision-making and self-efficacy, which affect their career development and enable them to adapt to the daily management and treatment of psychiatric patients. These actions will greatly influence their career advancement and enhance their ability to effectively manage and treat psychiatric patients on a regular basis.

The limitations of this study are as follows: This study selected psychiatric nursing students who practiced in our hospital within a specific time range, which may lead to limitations of the sample. Our study employed a single-point, convenience sampling, and cross-sectional design, which may limit the generalizability and generalization of the results. Additionally, our research focused solely on the relationship between career decision self-efficacy and maladaptation, without considering other potential influencing factors, which may render our conclusions relatively one-sided. Furthermore, our study utilized correlation and regression analysis methods, which, although capable of preliminary exploration of relationships between variables, cannot establish causal relationships. This study was conducted only in a specific healthcare setting, and the unique characteristics of that setting may limit the generalizability of the findings to other healthcare institutions with different backgrounds and nursing practices. Future research can compensate these limitations through multicenter, long-term studies with more refined designs and larger samples.

## Conclusion

Psychiatric nursing students experience maladjustment during clinical practice, and self-efficacy related to career decision-making is the main influencing factor of maladjustment during clinical practice.

## Availability of Data and Materials

Data are available from the corresponding author on reasonable request.
